# The Genetic Polymorphisms of CYP2C9 and VKORC1 in the Saudi Population and Their Impact on Anticoagulant Management

**DOI:** 10.3390/medicina61101872

**Published:** 2025-10-18

**Authors:** Mohammad Al Hamad

**Affiliations:** Department of Pathology, College of Medicine, Imam Abdulrahman Bin Faisal University, Dammam 31441, Saudi Arabia; mhamad@iau.edu.sa

**Keywords:** CYP2C9, VKORC1, warfarin therapy, Saudi population, therapeutic index

## Abstract

*Background and objectives*: Warfarin is a commonly used anticoagulant with a narrow therapeutic index that requires a precise dose to achieve efficacy and safety. Genetic variations in the CYP2C9 and VKORC1 genes significantly contribute to individual responses to warfarin, influencing both drug metabolism and pharmacodynamics. The current study aims to investigate the frequency of CYP2C9 and VKORC1 variant genotypes and determine the appropriate warfarin dosage for patients in Saudi Arabia. *Materials and Methods*: Blood samples were collected from 100 Saudi patients undergoing treatment with warfarin. DNA was extracted and purified from the whole blood, and variants in the CYP2C9 and VKORC1 genes were analyzed using multiplex PCR techniques. *Results*: The analysis revealed that the VKORC1 GG genotype was the most common, at 54%, followed by GA at 30%, and the AA at 16%. For CYP2C9, the *1/*1 genotype predominated at 71%, whereas the *1/*2 genotype was found in 14%, the *1/*3 genotype was found in 11%, and the *2/*3 genotype was found in in 2%, being less frequently observed. Patients with VKORC1 GG required significantly higher warfarin doses than those with GA and AA genotypes. Similarly, CYP2C9 *1/*1 patients required higher doses than those with *1/*3 and *2/*3 variants. No significant differences in INR levels across genotypes were found, indicating that while genetic variations influence dosing, they do not significantly alter the therapeutic INR range. *Conclusions*: The findings indicate that genetic variations influence drug metabolism and response in the Saudi population, aligned with global studies. Such tailored approaches could enhance treatment efficacy and reduce adverse effects, underscoring the role of pharmacogenomics in patient care and optimizing warfarin therapy in unique genetic populations.

## 1. Introduction

Warfarin is an anticoagulant medication utilized in the management of several medical conditions, including deep vein thrombosis, pulmonary embolism, myocardial infarction, and atrial fibrillation [1,2]. It has a narrow therapeutic index, necessitating individualized dose adjustment monitored by the international normalized ratio (INR), which is maintained within a therapeutic range of 2 to 3 [3]. An inappropriate dose, whether too high or low, can elevate the risk of bleeding or thrombosis, respectively [4,5]. The required dosage of warfarin is influenced by a range of factors, including age, body mass index (BMI), smoking, and gender, as well as genetic factors, particularly variations in CYP2C9 and VKORC1 genes [6,7,8].

Warfarin is provided as a racemic mixture consisting of an equal proportion of R-warfarin and S-warfarin. Notably, S-warfarin exhibits a greater potency compared to R-warfarin, and CYP2C9 exclusively metabolizes it [9]. CYP2C9 is categorized among the cytochrome P450 enzymes, which play a significant role in warfarin’s action, clearance, and metabolism [10,11]. Importantly, mutations in the CYP2C9 affect the metabolism and excretion of warfarin. Typically, patients who possess a CYP2C9 mutation require a lower dose of warfarin due to the reduced metabolic rate of this medication [1,12]. Individuals with CYP2C9*2 and/or CYP2C9*3 variant alleles can exhibit a decreased enzyme activity of up to approximately 70%, requiring a reduced concentration of warfarin [9]. The frequency of CYP2C9 alleles varies based on the patient’s ethnicity; Caucasians have the highest incidence of the CYP2C9*2 variant, while it is least prevalent among those with African descent. Furthermore, the CYP2C9*3 is less frequent in those African ethnicities, whereas variants such as CYP2C9*5 or CYP2C9*6 may be more frequently encountered [12,13]. Beyond warfarin, CYP2C9 polymorphisms affect the metabolism of other widely used drugs, underscoring the broader clinical utility of pharmacogenetic testing.

The Vitamin K epoxide reductase multiprotein complex (VKORC1) constitutes the principal enzyme responsible for regulating the vitamin K cycle [14]. A key component of this complex is Vitamin K epoxide reductase complex subunit 1 (VKORC1), which serves as the target enzyme for warfarin [15]. A frequently observed variant of this enzyme is the (VKORC1) 1639G>A polymorphism, which results in a decrease in the enzyme activity of VKORC1 [16]. Consequently, patients exhibiting (VKORC1) 1639G>A polymorphism require a reduced initial warfarin dose [9]. The -1639 genotype stands out as a critical determinant in establishing the appropriate dose of warfarin [12]. Many studies have reported a variation in the prevalence of CYP2C9 and VKORC1 variant alleles across ethnic groups, particularly among Asian and Indian populations. Thus, understanding a patient’s ethnicity is a significant factor in predicting the likelihood of such variant alleles [17,18]. The initial warfarin dose typically ranges from 2.5 to 5 mg daily, with 5 mg generally considered the standard for most adults. However, lower doses (2.5–3 mg) are recommended for elderly patients or those at high bleeding risk [19]. Maintenance doses are adjusted to achieve a target INR of 2.0–3.0. Typically, most patients require a daily dose ranging from 2 to 10 mg (average 4–5 mg) [2]. Dose adjustments should be guided by frequent INR monitoring, particularly during initiation, with consideration for pharmacogenomic factors (CYP2C9/VKORC1 variants) and drug interactions [20].

Considering the polymorphisms of CYP2C9 and VKORC1 genes, the FDA has sanctioned a warfarin label designed to aid in determining the initial maintenance dose of warfarin [21]. Furthermore, patients carrying a variant allele will necessitate more frequent monitoring of INR testing to mitigate the risk of potential complications [22]. The inappropriate administration of warfarin doses poses significant risks, including serious adverse effects such as bleeding events or embolism, which could ultimately result in fatality [23]. Furthermore, multi-gene dosing algorithms that incorporate additional pharmacogenetic factors, such as polymorphisms in the CYP4F2 gene, alongside clinical variables, have been developed to refine warfarin dose prediction, demonstrating superior accuracy compared to clinical dosing alone [24]. Research indicates that if the healthcare providers had performed genomic testing before selecting and administering the warfarin dose, it is conceivable that approximately 85,000 cases of bleeding and 17,000 cases of stroke may not have occurred [25]. While regional data is emerging, studies from neighboring Middle Eastern populations, such as Kuwait, Egypt, and Oman, show notable variations in the prevalence of these key polymorphisms [26,27,28,29]. This underscores the necessity for precise, population-specific data rather than relying on broad ethnic categorizations. The Gage/IWPC (International Warfarin Pharmacogenetics Consortium) methods that incorporate clinical variables (age, weight, interacting drugs) and genetic variants in CYP2C9 and VKORC1. CPIC (Clinical Pharmacogenetics Implementation Consortium) provides standardized, evidence-based guidelines on how to use genetic test results to guide warfarin therapy. These are clinically validated tools that enhance the safety and efficacy of warfarin therapy. They have been shown to lower out-of-range INRs and adverse events in various populations [30]. Real-world application in Middle Eastern populations validates their general utility while showcasing an important difference: European-derived algorithms frequently over-predict dose levels due to a high prevalence of the VKORC1 low-dose haplotype [31,32,33]. Thus, while genotype-driven dosing remains useful for preventing initial overdosing in the Middle East, achieving optimal results relies on context-specific validation and cohort-based algorithms that target the unique genetic landscape of the region.

This study’s primary objective is to assess the prevalence of CYP2C9 and VKORC1 variant genotypes in the eastern province of the Saudi Arabian population and to analyze how these genetic polymorphisms affect warfarin dosage tailored to the specific CYP2C9 and VKORC1 variants present in the Saudi population.

## 2. Materials and Methods

### 2.1. Patients and Study Design

This cohort study compares patient groups based on genetic makeup and monitors their response to warfarin. A list of all eligible Saudi patients undergoing warfarin treatment at the anticoagulation clinic of King Fahd University Hospital was generated from the hospital database. From this list, 100 patients were randomly selected using a computer-generated random number. The study included patients over 18 years of average age who had cardiovascular disease. However, patients with mechanical valves, acute thromboembolic events, or active liver disease at the time of anticoagulant initiation were excluded from this study. Using our hospital’s patient database, clinical and laboratory data, including the INR, as well as the mean weekly warfarin dose, were utilized to adjust the patient’s warfarin dose. INR testing was conducted at King Fahd University Hospital in accordance with the College of American Pathologists (CAP) guidelines to ensure standardized quality and accuracy.

This study focused on high-impact allelic variants—CYP2C9*2 (rs1799853), *3 (rs1057910), and VKORC1-1639 G>A (rs9923231)—based on CPIC and FDA pharmacogenetic guidelines. These well-established markers form the core of warfarin dosing algorithms and provide a clinically robust foundation for evaluating genetic influences on warfarin response in the Saudi population. Other variants, like CYP4F2 variants, were excluded due to their smaller effect sizes and limited relevance to this initial analysis.

After receiving informed consent, blood samples (EDTA) were collected from selected patients at King Fahd University of Hospital. DNA was extracted from the whole blood samples using the Qiamp DNA Blood Mini Kit (Qiagen, Germany). DNA was purified using Qiacube from Qiagen, a fully automated system. A volume of 50 µL was used for each sample as per the manufacturer’s recommendations. DNA quantity and purity were assessed using Poch-Bioteck (Santa Clara, USA). DNA integrity was assessed using the QIAxcel Advanced System (Qiagen), which provided high-resolution separation and minimal degradation. The concentration of DNA was quantified in nanograms per microliter (ng/μL), calculated using the formula A260 × 50 × Dilution Factor specifically for double-stranded DNA (dsDNA). Additionally, the assessment of purity was conducted through the A260/A280 ratio, where a value ranging from 1.8 to 2.0 signifies the presence of pure DNA.

### 2.2. Polymerase Chain Reaction (PCR)

In summary, PCR amplification was performed in a reaction volume of 20 µL, which consisted of 2 µL of genomic DNA incorporated into 18 µL of the appropriate MasterMix for either CYP2C9*2 (430C>T), CYP2C9*3 (1075A>C), or VKORC1 (1639G>A), all placed in PCR tubes. Each reaction included a wild-type positive control, a positive control for the homozygous mutant, a positive control for the heterozygous mutant, a negative control, and samples from patients. The detection kit utilizes “hot start” technology, minimizing non-specific reactions and assuring maximum sensitivity. The thermal cycling process was conducted using a Rotor-Gene TM 3000 Q machine (Hilden, Germany), with an initial denaturation step at 95 °C for 10 min, followed by 40 cycles at 95 °C for 10 s, with the annealing temperature maintained at 64 °C for 20 s, and an extension step at 72 °C for 20 s, before the final extension at 72 °C for 5 min.

Validation of the multiplex PCR assay for VKORC1 and CYP2C9 genotyping involved replicate testing, strict controls, and orthogonal confirmation. Samples were analyzed in duplicate and across different days to assess intra- and inter-assay variability. Controls included DNA samples with known wild-type and variant genotypes for VKORC1 (1639G>A) and CYP2C9 (*2/*3 alleles), no-template controls (NTC), and an internal amplification control (β-globin). Genotype accuracy was confirmed using Sanger sequencing.

### 2.3. VKORC1 and CYP2C9 Variants

**Figure 1:**
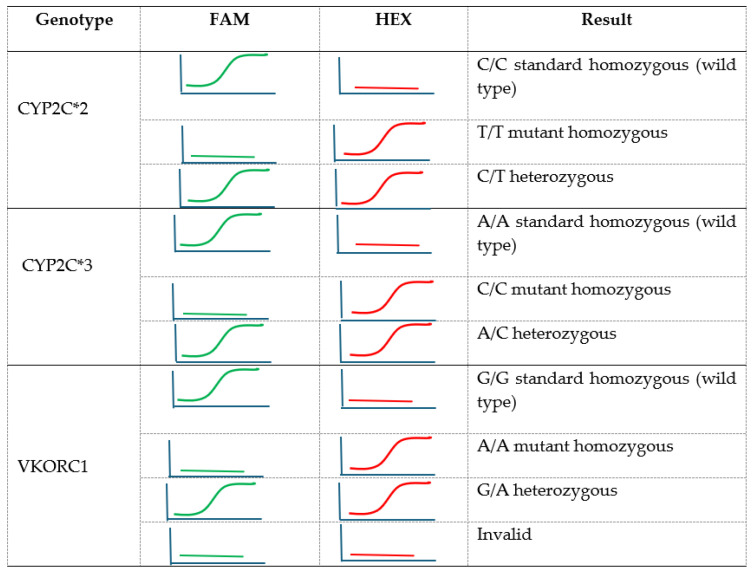
This figure represents the interpretation of CYP2C9 and VKORC1 Variants by multiplex real-time PCR.

Detection of VKORC1 and CYP2C9 variants is based on amplification and detection of the target sequence using allele-specific fluorophore-labeled probes. Target sequences are in the VKORC1 (G1639A) gene and the allelic variants CYP2C9*2 (C430T) and CYP2C9*3 (A1075C) of the gene encoding cytochrome P450 2C9. The warfarin Detection Kit was employed with the Rotor-Gene Q, designed for real-time and end-point thermal cycling using the polymerase chain reaction (PCR) and high-resolution melting analysis (HRM™). Wild-type alleles are detected on the FAM fluorescent channel and mutant alleles on the HEX fluorescent channel. However, the heterozygote is detected on both channels. Results interpretation details are presented in Figure 1:

### 2.4. Statistical Analysis

The statistical examination for this research utilized Minitab (version 21.2) to assess the connection between genetic polymorphisms and warfarin dosage needs in the Saudi population. Descriptive statistics were generated to outline the genotype frequencies for CYP2C9 and VKORC1, as well as the associated distributions of warfarin dosages. Additionally, regression analysis was performed to assess how particular genotypes affect the variability in weekly warfarin doses.

## 3. Results

At King Fahd Hospital of the University, 100 blood samples were collected from patients with cardiovascular disease. 71 males (71%) and 29 females (29%) participated. The patients’ ages ranged from 20 to 88, with a median age of 57.

### 3.1. CYP2C9 and VKORC1 Genotypes

The distributions of CYP2C9 and VKORC1 genotypes in our cohort are detailed in Table 1. Notably, the wild-type genotypes CYP2C9 *1/*1 (71%) and VKORC1 GG (54%) were the most prevalent. Although CYP2C9 and VKORC1 genotypes have been reported to have a close correlation, our findings demonstrated a higher incidence of discordance compared to two studies from Japan and China [9,34]. This would need further exploration as it may suggest variations in linkage disequilibrium (LD) patterns between different ethnic groups.

Our results demonstrate that the three CYP2C9 variants and VKORC1-1639 variant alleles were the most prevalent in our cohort of patients, comprising 90% of total genotypes. CYP2C9*1, *2, and *3 allele frequencies were 83%, 9%, and 8%, respectively, while the VKORC1-1639 variant allele frequency for G was 69%. [35] Table 1.

### 3.2. Association Between Genotypes and Clinical Outcomes

The association between CYP2C9 and VKORC1 genotypes and the INR in patients from the Saudi population was evaluated. We also investigate how different genotypes affect the stable dose of warfarin. Patients with the CYP2C9*2 or CYP2C9*3 genotypes and VKORC1 AA or VKORC1 GA required a significantly lower mean daily and weekly warfarin dose to achieve stable anticoagulation. In contrast, those with the CYP2C9*1*1 and VKORC1 GG genotypes required a substantially higher dose. The mean weekly warfarin dose in VKORC1 GG, VKORC1 AA, CYP2C9*2, and CYP2C9*3 heterozygotes, and CYP2C9*2 homozygotes was significantly lower than that in CYP2C9*1*1, VKORC1 GG patients (*p* = 0.00001). However, the effect of both CYP2C9 and VKORC1 genotypes on INR response was insignificant Table 2. Multiple linear regression analysis revealed significant genotype–dose associations after adjusting for age and gender. The model showed excellent fit (R^2^ = 0.48, *p* < 0.001) with no evidence of multicollinearity (all VIFs < 2). *VKORC1* variants demonstrated the strongest effects: the GG genotype required 6.23 mg/day more than AA (95% CI: 4.36–8.10, *p* < 0.001), while GA required 3.11 mg/day more (95% CI: 1.29–4.93, *p* = 0.001). Among *CYP2C9* variants, only *1/*3 showed significant dose reduction (−3.55 mg/day, 95% CI: −5.79 to −1.31, *p* = 0.002). Male gender was associated with lower doses (−1.97 mg/day, 95% CI: −3.59 to −0.35, *p* = 0.018), while age showed no significant effect (*p* = 0.203). These results demonstrate that genetic factors account for substantial dose variability independent of demographic characteristics, with *VKORC1* GG carriers requiring approximately 150% higher doses than AA genotypes, supporting genotype-guided warfarin dosing in clinical practice Table 3.

**Figure 2:**
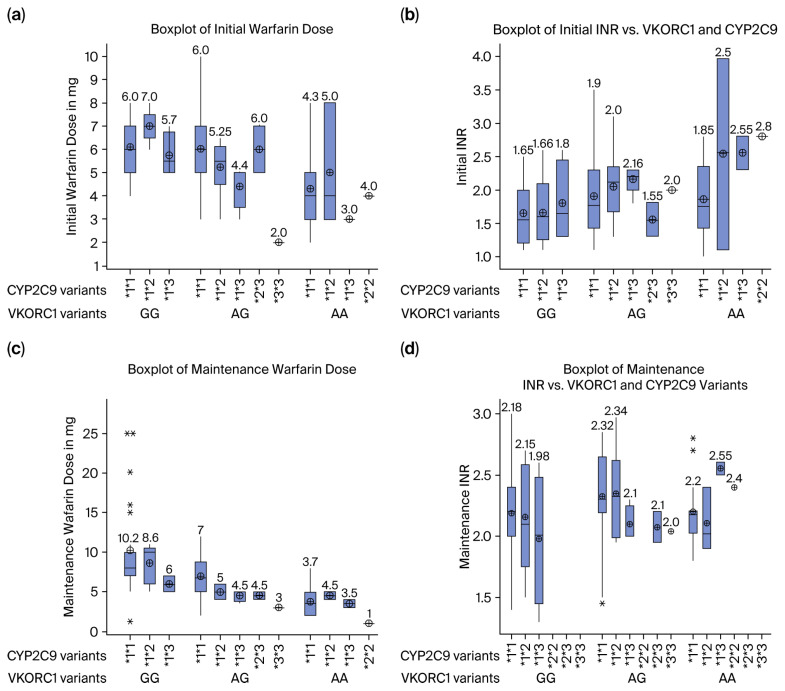
Presents boxplots comparing daily warfarin doses and corresponding INR levels across different VKORC1 and CYP2C9 genotypes. Panels (**a**,**b**) show initial doses and INR responses, revealing how genetics influence early therapy, while panels (**c**,**d**) display maintenance doses and stable INR levels after personalized dose adjustment. Stars denote outliers who required exceptionally high doses. The figure demonstrates that while genetic variants necessitate different warfarin doses, proper titration achieves equivalent therapeutic INR control.

We compared the initial and maintenance daily warfarin dose with their corresponding INR levels against different VKORC1 and CYP2C9 genotype variants. The data show that initial doses are typically higher and more variable, aiming to achieve therapeutic INR (2–3), while maintenance doses are lower and personalized based on genetic sensitivity. For example, VKORC1 AA and CYP2C9 *3*3 carriers require significantly lower doses (both initial and maintenance) due to heightened warfarin sensitivity, often resulting in higher INR values if not carefully titrated. Conversely, *VKORC1 GG* and CYP2C9 *1*1 patients require higher doses to achieve and maintain therapeutic INR levels. The boxplots highlight genotype-driven dose disparities and underscore the importance of pharmacogenomics in optimizing warfarin therapy to avoid bleeding (high INR) or clotting (low INR) risks during both phases. Figure 2. In comparison of the daily average warfarin dose across different populations, our study reveals that Saudi patients with VKORC1 GG and CYP2C9 *1/*1 genotypes require higher doses (10.6 mg/day) compared to the FDA guideline range (5–7 mg/day), Table 4 [36], which reinforces the importance of pharmacogenetic testing for precise anticoagulant management. It is important to highlight that the five patients with the CYP2C9 *1*1 and VKORC1 GG genotypes were identified as outliers, requiring 15–25 mg/day to achieve the target INR (Figure 2).

### 3.3. The Impact of VKORC1 and CYP2C9 Genotype Variants on the Weekly Average Warfarin Dose

As shown in Table 5, patients with the VKORC1 GG genotype required a significantly higher average weekly warfarin dose than those with GA or AA genotypes (*p* < 0.00001), a trend that was also observed for CYP2C9 *1/*1 carriers compared to those with variant alleles. Similarly, for the CYP2C9 *1 *1 wild genotype, the average dosage of 54.14 mg surpasses that of the CYP2C9 *1 *2, *1 *3, *2, and *2 *3 variants, as presented in Table 5.

The median weekly dosage of warfarin for the cohort comprising patients with the CYP2C9 *1*1 and VKORC1 GG genotypes was 62.25 mg/week. This contrasts with the dosages for patients harboring the CYP2C9 *1*1, VKORC1 GA and CYP2C9 *1*1, VKORC1 AA genotypes, which were 49 mg/week and 22 mg/week, respectively; see Table 5.

### 3.4. Genetic Diversity in VKORC1 and CYP2C9 Among Different Ethnicities

The distribution of genetic variants in VKORC1 and CYP2C9 varies significantly among ethnic groups, as detailed in Table 6. Our cohort from the Eastern Province of Saudi Arabia demonstrates a distinct genetic profile, characterized by a high frequency of the VKORC1 GG genotype (54%) and the CYP2C9 *1/*1 genotype (71%). This stands in stark contrast to East Asian populations (e.g., 80% VKORC1 AA in Chinese) and differs notably from neighboring Middle East populations like Kuwait, Egypt, and Oman, which show a higher prevalence of VKORC1 GA genotype. Even within the Middle East, our findings reveal substantial heterogeneity, underscoring that the Saudi population cannot be grouped under a single ‘Middle Eastern’ pharmacogenetic profile. These regional disparities form the genetic basis for the observed differences in warfarin dosing requirements.

## 4. Discussion

The results of this investigation establish that the genetic architecture underlying warfarin response in the Saudi population from the Eastern Province is distinct, characterized by a high prevalence of the VKORC1 GG and CYP2C9 *1/*1 genotypes. This genetic profile directly translates into a significantly higher warfarin dose requirement compared to populations used to establish international dosing guidelines. Our study highlights distinct genetic patterns in the Saudi population, with a high prevalence of the VKORC1 GG genotype (54%) and the CYP2C9 *1/*1 genotype (71%), compared to other studies from different provinces in Saudi Arabia [42,43]. Furthermore, other Middle Eastern and North African populations, such as Egypt, Kuwait, Oman, Bahrain, and Tunisia [26,27,28,29,40,41], exhibit a higher frequency of the VKORC1 GA genotype (51%, 50% 42.7%,41%, 66%, respectively) and moderate CYP2C9 *1/*2 (19–21%). Turkey stands out with extreme GA dominance (75.4%) and elevated CYP2C9 *1/*2 (23%). Crucially, these genetic disparities translate directly to clinical practice [39].

When our data are applied to the CPIC dosing algorithm [36], the recommended starting dose for a typical Saudi patient (VKORC1 GG, CYP2C9 *1/*1) is 5–7 mg/day. However, our cohort required a mean daily dose of 10.6 mg to achieve therapeutic anticoagulation (Table 4). This systematic underdosing, if applied clinically, would place a significant proportion of patients at sub-therapeutic INR levels and increased risk of thromboembolism. This discrepancy provides a compelling argument for developing population-specific dosing algorithms. The regional differences underscore the importance of population-specific pharmacogenetic testing to optimize anticoagulant therapy, as generalized dosing guidelines may not account for genetic diversity observed across the Middle East. This prevalence diverges from that observed in other ethnic groups, such as East Asians, where the VKORC1 AA genotype is the most frequent (80%), and Caucasians, who show an increased occurrence of CYP2C9 *1/*2 and *1/*3 variants. Such disparities underscore the need for pharmacogenetic data tailored to specific populations, as genetic variability significantly impacts warfarin dosing protocols. These observations reaffirm the established roles of CYP2C9 and VKORC1 in modulating warfarin metabolism and sensitivity, accentuating the importance of employing genotype-guided dosing strategies. The finding that INR values did not differ significantly across genotypes, despite substantial variations in warfarin dose requirements, can be attributed to the fundamental principle of warfarin therapy: the dose is actively titrated to achieve a specific target INR, thereby normalizing the anticoagulant effect across different genetic profiles once a stable maintenance dose is established. This convergence likely masks the initial challenges of dose-finding and underscores the critical role of continuous clinical monitoring to manage confounding factors. A more nuanced explanation must consider that dietary fluctuations in vitamin K intake, variable medication adherence, and the presence of comorbidities or interacting drugs can introduce significant INR variability that obscures any subtle, direct genotype-INR relationship. Therefore, the primary impact of CYP2C9 and VKORC1 polymorphisms is demonstrated not in the final therapeutic INR, but in the vastly different dose required to reach it, highlighting the value of genotyping for guiding initial dosing to achieve a faster time in therapeutic range and improve safety during the critical initiation phase. The study’s hypotheses were largely confirmed by the data, asserting that the genetic polymorphisms within CYP2C9 and VKORC1 substantially influence warfarin dosing among the Saudi population. Furthermore, the frequency of these polymorphisms is distinct when compared to other ethnic groups, necessitating customized dosing recommendations. The potential for pharmacogenetic testing to enhance warfarin management, mitigate adverse effects like bleeding or thrombosis, and ultimately improve patient outcomes in Saudi Arabia is underscored by these findings. The results advocate for region-specific clinical guidelines, as generalized recommendations based on Caucasian or East Asian studies may not be directly transferable to Middle Eastern populations. The adoption of genotype-guided dosing strategies could lower the healthcare costs associated with warfarin-related complications, supporting prior estimates indicating that pharmacogenetic testing could avert thousands of cases of bleeding and stroke each year. The implications of our findings extend beyond optimizing warfarin therapy. The CYP2C9 enzyme is responsible for the metabolism of a wide range of clinically important drugs. The high prevalence of the wild type *1/*1 genotype (71%) in our cohort suggests that most of the Saudi population are normal metabolizers for CYP2C9 substrates. However, 29% of individuals carrying variant alleles (*2, *3) are at risk for altered drug metabolism. For example, Patients with CYP2C9*3 variants require significantly lower doses of Phenytoin to avoid drug accumulation and toxicities such as nystagmus, ataxia, and drowsiness [44]. Individuals identified as poor metabolizers of CYP2C9 may experience a reduced conversion of losartan to its active metabolite, potentially diminishing its antihypertensive effect. Drugs like ibuprofen and celecoxib are also metabolized by CYP2C9. Poor metabolizers may have an increased risk of gastrointestinal bleeding and cardiovascular events due to higher drug exposure [45]. Therefore, the pharmacogenetic data presented here for the Saudi population is not only critical for anticoagulation management but also provides a valuable foundation for personalizing treatment with a broad spectrum of therapeutic agents, improving drug safety and efficacy across multiple clinical disciplines. An analysis of the patient cohort revealed that five individuals (5 out of 26) exhibited wild-type genotypes for both VKORC1 and CYP2C9. These patients required warfarin dosages ranging from 15 mg to 25 mg, as illustrated in Figure 2c. It is important to consider that these patients may possess alternative genetic variants, such as those associated with CYP4F2, as well as various non-genetic factors that could influence warfarin metabolism. Extending this research to encompass a broader and more diverse Saudi population may further substantiate these conclusions and uncover additional genetic or environmental factors that influence warfarin response, particularly among patients requiring more than 15 mg of warfarin per day. Future inquiries might delve into the roles of other genetic factors, such as CYP4F2 and CALU, alongside non-genetic variables that contribute to individual variability in warfarin response [43]. Furthermore, recent studies like the Saudi Warfarin Pharmacogenetic (SWAP) study highlight the significant role of additional genes, including CYP2C19 in alternative warfarin metabolism and CYP2C8 in vitamin K metabolism, which can further refine dose predictions. Crucially, population-specific research continues to identify novel variants, underscoring that the full spectrum of genetic determinants of warfarin response is both complex and ethnically diverse [46]. Such comprehensive methodologies could significantly enhance personalized medicine initiatives, leading to improved patient safety and treatment efficacy.

While pharmacogenomics provides a powerful tool for personalizing therapy, it is essential to integrate the non-genetic measures for proper dose adjustment. Genotype-guided dosing should be viewed as the starting point for a dynamic and ongoing process. Key non-genetic factors that necessitate continuous monitoring and adjustment include clinical parameters such as patient age, body weight, and comorbidities (especially liver and renal function), which significantly influence drug pharmacokinetics and response. Concomitant administration of medications that inhibit (e.g., amiodarone, fluconazole) or induce (e.g., rifampin, carbamazepine) CYP enzymes can drastically alter warfarin metabolism, overriding a patient’s genetic profile [47]. Fluctuations in the intake of vitamin K-rich foods (e.g., leafy green vegetables) can cause significant variations in INR, requiring dose modifications. Therefore, the most effective approach to anticoagulant management is a holistic one that combines preemptive genotyping with vigilant clinical assessment and monitoring, ensuring both the precision of the initial dose and the safety of long-term therapy.

While this study provides valuable insights into the pharmacogenetics of warfarin in the Eastern province of the Saudi population, certain limitations must be considered—the sample size of 100 patients. Although sufficient for identifying the predominant effects of common genotypes like VKORC1 GG and CYP2C9 *1/*1, this cohort provides limited statistical power for robust analysis of rare variant associations. Consequently, the estimated warfarin dose requirements for these rare genotypic groups should be interpreted with caution.

## 5. Conclusions

This study contributes to the existing evidence supporting the use of pharmacogenetics in improving warfarin therapy. The distinct genetic characteristics of the Saudi population necessitate customized methods for managing anticoagulant treatment. It also ensures that warfarin therapy is safer and more effective with a clinical impact. Although the centralized nature of the existing healthcare system and the laboratory infrastructure support its clinical feasibility for successful implementation, there is a need to further enhance clinician education, local guidelines, and insurance coverage to make testing cost-effective. Addressing these challenges is crucial to translating this personalized medicine approach into routine practice, improving patient safety and outcomes. Further studies should aim to refine dosing algorithms and broaden their application to include various populations and newer anticoagulant medications.

## Figures and Tables

**Table 1:** The frequency of VKORC1 1639 G>A and CYP2C9 *2 *3 genotype alleles in the patients’ samples.

Genotype and Alleles	No. of Patients (Frequency%)
VKORC1 genotype and alleles	
GG	54 (54%)
AG	30 (30%)
AA	16 (16%)
G	69%
A	31%
CYP2C9 genotype and alleles	
*1/*1	71 (71%)
*1/*2	14 (14%)
*2/*2	1 (1%)
*1/*3	11 (11%)
*2/*3	2 (2%)
*3/*3	1 (1%)
*1	83%
*2	9%
*3	8%

**Table 2:** The INR means in patients with genotype variants of CYP2C9 and VKORC1.

Genotypes and Alleles	Number	Mean of INR	95% CI	*p* Value
CYP2C9 *1*1	71	2.436	(2.115, 2.757)	
CYP2C9 *1*2	14	2.226	(1.507, 2.944)	
CYP2C9 *1*3	11	2.138	(1.328, 2.949)	
CYP2C9 *2*2	1	2.400	(−0.288, 5.088)	0.976
CYP2C9 *2*3	2	2.075	(0.174, 3.976)	
CYP2C9 *3*3	1	2.040	(−0.648, 4.728)	
VKORC1 GG	54	2.2805	(2.099, 3.005)	
VKORC1 GA	30	2.2805	(1.8777, 2.6832)	
VKORC1 AA	16	2.2264	(1.6633, 2.7894)	0.584

**Table 3:** Regression analysis: maintenance warfarin dose versus Age, gender, VKORC1, and CYP2C9.

Term	Coef	SE Coef	T-Value	*p*-Value	VIF
Constant	7.05	1.47	4.78	0.000	
Age	−0.0285	0.0222	−1.28	0.203	1.05
VKORC1 variants					
GA	3.111	0.919	3.39	0.001	1.83
GG	6.234	0.949	6.57	0.000	1.82
CYP2C9 variants					
*1*2	−1.568	0.991	−1.58	0.117	1.05
*1*3	−3.55	1.13	−3.15	0.002	1.10
*2*2	−4.22	3.49	−1.21	0.229	1.07
*2*3	−2.19	2.45	−0.89	0.373	1.04
*3*3	−4.08	3.43	−1.19	0.238	1.03
Gender					
Male	−1.971	0.819	−2.41	0.018	1.17

**Table 4:** The distribution of the average daily dosage of warfarin across populations.

Genotype	Present Study (Saudi Patients) (mg/day)	FDA Guidelines (mg/day) [36]
VKORC1 GG and CYP2C9 *1/*1	10.6	5–7
VKORC1 GG and CYP2C9 *1/*2, *1/*3	6.2–7.3	3–4
VKORC1 GG and CYP2C9 *2/*2, *2/*3, *3/*3	0.5–2	0.5–2
VKORC1 GA and CYP2C9 *1/*1	7.4	5–7
VKORC1 GA and CYP2C9 *1/*2, *1/*3	5.1–5.6	3–4
VKORC1 GA and CYP2C9 *2/*2, *2/*3, *3/*3	0.5–2	0.5–2
VKORC1 AA and CYP2C9 *1/*1	4.2	3–4
VKORC1 AA and CYP2C9 *1/*2, *1/*3	3.4–3.8	0.5–2
VKORC1 AA and CYP2C9 *2/*2, *2/*3, *3/*3	0.5–2	0.5–2

**Table 5:** Average weekly doses of warfarin in patients with various VKORC1 and CYP2C9 genotype variants.

Gene Variants	No. of Patients (Frequency %)	Average Weekly Warfarin Initial Dose (mg)	*p*-Value
VKORC1 GG	54 (54%)	68.47	
VKORC1 GA	30 (30%)	45.05	
VKORC1 AA	16 (16%)	25.88	*p* < 0.00001
CYP2C9 *1/*1	71 (71%)	54.2	
CYP2C9 *1/*2	14 (14%)	42.4	
CYP2C9 *1/*3	11 (11%)	34	
CYP2C9 *2/*3	2 (2%)	28	
CYP2C9 *2/*2, CYP2C9 *3/*3	2 (2%)	18	*p* < 0.0357
VKORC1 GG CYP2C9 *1/*1	26 (26%)	74.4	
VKORC1 GG CYP2C9 *1/*1, *1/*2, *2/*3, *2/*2, *3/*3	9 (9%)	51.3	
VKORC1 GA CYP2C9 *1/*1	29 (29%)	51.4	
VKORC1 GA, CYP2C9 *1/*2, *2/*3, *2/*2, *3/*3	14 (14%)	31.9	*p* < 0.00001
VKORC1 AA CYP2C9 *1*1	16 (16%)	25.2	
VKORC1 AA, CYP2C9 *1/*2, *2/*3, *2/*2, *3/*3	5 (5%)	24.7	

**Table 6:** The frequency of VKORC1 and CYP2C9 genotype variants in different geographic groups.

Race	VKORC1			CYP2C9					
	GG	GA	AA	*1*1	*1*2	*2*2	*1*3	*2*3	*3*3
Caucasian [37,38]	39%	47%	14%	67%	17%	3%	13%	0%	0%
UK [6]	25%	56%	19%	56%	22%	3%	14%	4%	<1%
Japan [9]	23%	58%	19%	23%	4%	0%	51%	17%	5%
Turkey [39]	21.9%	75.4%	2.7%	56.5%	23%	3%	8.6%	7.2%	1.7%
Chinese [34]	2**%**	18**%**	80**%**	91**%**	0**%**	0**%**	**7%**	1.5**%**	0**%**
India [1]	69**%**	27**%**	4**%**	74**%**	8**%**	0**%**	16**%**	1.5**%**	0.05%
Egypt [26,27]	24%	51%	25%	66%	19%	2%	11%	0%	4%
Tunisia [40]	9.3%	66%	24%	62%	19%	3%	13%	3%	0%
Kuwait [28]	35.2%	50%	14.8%	69.4%	21.3%	0%	6.5%	2.8%	0%
Oman [29]	42.7	42.7	14.6	75%	11.5%	0%	8.7%	2.4%	1%
Bahrain [41]	43.6%	41.1%	15.25	68.2%	18.2%	1.7%	10.6	0.8%	0.4%
Present study	54%	30%	16%	71%	14%	1%	11%	2%	1%

## Data Availability

Anonymized data supporting this study’s findings are available from the corresponding author upon reasonable request.

## Abbreviations

The following abbreviations are used in this manuscript:

INR	International normalized ratio
PCR	Polymerase chain reaction
LD	linkage disequilibrium
BMI	Body mass index
VKORC1	Vitamin K epoxide reductase multiprotein complex
HRM	high-resolution melting analysis
NTC	No-template controls
